# Clinical Midwifery

**Published:** 1849-11

**Authors:** 


### ARTICLE VI.

*Clinical Midwifery. Comprising the Histories of Five Hundred and Forty-five Cases of Difficult, Preternatural, and Complicated Labor. With Commentaries.*—By ROBERT LEE, M.D., F.R.S., Fellow of the Royal College of Physicians, London; Physician to the British Lying-in Hospital and St. Marylebone Infirmary; Lecturer on Midwifery at St. George's Hospital. First American from the Second London Edition. Philadelphia: Lea & Blanchard. 1849. pp. 238. 12 mo. (From the Publishers. For sale by S. C. Griggs & Co., Chicago.)

This little volume, as its name implies, is entirely taken up with a detail of cases of preter-natural labor. From the peculiar position of the Author, a great number of this description of cases would naturally come under his care; and as every physician should do, he has by keeping a careful record, rendered his great experience subservient to the advantage of his professional brethren, who, although occupying less conspicuous positions, would occasionally meet with a difficult case of labor. Books of this kind present great attractions to the practical man: and serve to show the importance of preserving a written record of all interesting and unusual cases. In this way a physician accumulates materials for a book of the very best kind, and one which is a better substitute for clinical instruction, than anything else we know of.

The cases detailed, extend over a period of fifteen years in the author's experience, and embrace every variety of operative Midwifery, except Hysterotomy, which he seems never to have performed. This little volume is a valuable one, and worthy a place in the library of every practitioner. M.

